# The prevalence and genomic characteristics of hepatitis E virus in murine rodents and house shrews from several regions in China

**DOI:** 10.1186/s12917-018-1746-z

**Published:** 2018-12-22

**Authors:** Wenqiao He, Yuqi Wen, Yiquan Xiong, Minyi Zhang, Mingji Cheng, Qing Chen

**Affiliations:** Department of Epidemiology, School of Public Health, Guangdong Provincial Key Laboratory of Tropical Disease Research, Southern Medical University, 1838 Guangzhou North Road, Guangzhou, 510515 China

**Keywords:** Genomic characteristic, Hepatitis E virus, House shrew, Murine rodent, Prevalence

## Abstract

**Background:**

Urban rodents and house shrews are closely correlated in terms of location with humans and can transmit many pathogens to them. Hepatitis E has been confirmed to be a zoonotic disease. However, the zoonotic potential of rat HEV is still unclear. The aim of this study was to determine the prevalence and genomic characteristics of hepatitis E virus (HEV) in rodents and house shrews.

**Results:**

We collected a total of 788 animals from four provinces in China. From the 614 collected murine rodents, 20.19% of the liver tissue samples and 45.76% of the fecal samples were positive for HEV. From the 174 house shrews (*Suncus murinus*), 5.17% fecal samples and 0.57% liver tissue samples were positive for HEV. All of the HEV sequences obtained in this study belonged to *Orthohepevirus* C1. However, we observed a lower percentage of identity in the ORF3 region upon comparing the amino acid sequences between *Rattus norvegicus* and *Rattus losea*. HEV derived from house shrews shared a high percentage of identity with rat HEV. Notably, the first near full-length of the HEV genome from *Rattus losea* is described in our study, and we also report the first near full-length rat HEV genomes in *Rattus norvegicus* from China.

**Conclusion:**

HEV is prevalent among the three common species of murine rodents (*Rattus. norvegicus*, *Rattus. tanezumi*, and *Rattus. losea*) in China. HEV sequences detected from house shrews were similar to rat HEV sequences. The high identity of HEV from murine rodents and house shrews suggested that HEV can spread among different animal species.

**Electronic supplementary material:**

The online version of this article (10.1186/s12917-018-1746-z) contains supplementary material, which is available to authorized users.

## Background

Hepatitis E virus (HEV) is the causative agent of hepatitis E in humans worldwide. According to 2018 data from the World Health Organization (WHO), there are 20 million HEV infections each year, leading to about 3.3 million symptomatic cases, with approximately one-third of the world’s population having been exposed to HEV [1]. HEV can cause hepatitis outbreaks and sporadic hepatitis, which is usually a self-limiting disease [2]. However, in immunosuppressed patients, HEV infection can cause rapidly progressive cirrhosis [3]. In the general population, the mortality rates of HEV infection range from 0.2–1%; however, the mortality rate is higher during pregnancy, especially in developing countries [4]. Therefore, HEV infection is a major global public health concern.

HEV is a positive-sense, single-stranded, non-enveloped RNA virus with a size of 27–34 nm. The genome of HEV contains three overlapping open reading frames (ORF), specifically ORF1 that encodes nonstructural proteins; ORF2 that encodes viral capsid protein, which is responsible for self-assembly of virus particles [5]; and ORF3 that encodes proteins involved in virus morphogenesis and release [6]. ORF4 has been identified in rat and ferret HEV strains; however, its function is still unknown [7–9].

HEV is classified in the genus *Orthohepevirus* in the family *Hepeviridae* [10]. *Orthohepevirus* is divided into four species, as follows: *Orthohepevirus* A, B, C, and D. All of the HEV strains isolated from humans belong to the species *Orthohepevirus* A with four genotypes, genotypes 1 and 2 can only infect humans, while genotypes 3 and 4 can infect animals and are considered zoonotic pathogens [11]. *Orthohepevirus* B includes avian HEV strains. *Orthohepevirus* C is divided into two species, one of which is *Orthohepevirus* C1, derived from rats, and the other of which is *Orthohepevirus* C2, derived from ferrets. Bat HEV is classified in *Orthohepevirus* D [2].

The origin of HEV is still unknown. HEV was first considered to be restricted to presentation in humans [12]. The most common transmission route of HEV is the fecal–oral route. However, in the recent years, HEV had been detected in a variety of animal species, such as pigs, wild boars, cattle, cats, rabbits, rodents, and dogs [13]. The consumption of meat or meat products and direct or indirect contact with pigs can be important modes of zoonotic HEV transmission [2, 11]. Hepatitis E has been confirmed to be a zoonotic disease [14]. In Japan, HEV strains isolated from *Rattus norvegicus* (*R. norvegicus*) that had been trapped near a pig farm where HEV was prevalent were genetically identical to the HEV strains derived from pigs [15], indicating that rats may be a reservoir for the HEV that causes infection in pigs.

Rat HEV was first detected in *R. norvegicus* from Germany [8]. Many studies since have reported the detection of HEV in rats. However, it is still inconclusive whether rat HEV can cross transmit to other species. In recent years, HEV has been detected in house shrews (*Suncus murinus*) trapped in China with a high similarity to the rat HEV strains, indicating that rat HEV might be transmitted between rats and shrews [16].

Murine rodents and house shrews are reservoirs of some human pathogens, such as mammarenavirus and hantavirus, owing to their close contact with humans [16]. However, the zoonotic potential of rat HEV is still unclear. Nonetheless, some studies have pointed out the possibility of HEV transmission between humans and rats. For example, recent investigations have shown that HEV genotype 3 has been detected in wild rats; rat HEV can successfully replicate in three human hepatoma cell lines (PLC/PRF/5, HuH-7, and HepG2 cells); and anti-rat HEV antibodies were detected in forest workers [15, 17–19].

It is important to understand the possibility of interspecies transmission of HEV between humans, rodents, and shrews. In China, the first HEV in rats was reported in 2013 [20]. So far, the presence of HEV in rats and shrews has only been reported in the Guangdong and Yunnan Provinces of China [16, 21]. Separately, the detection of full-length genomes of HEV in the Chevrier’s field mouse (*Apodemus chevrieri*) and Père David’s vole (*Eothenomys melanogaster*) have been reported in China [21]. However, the genomic characteristics of HEV in commensal rodents in China remain unclear.

In the present study, we investigated the prevalence of HEV in murine rodents and house shrews in five regions of four provinces in China. Besides, we analyzed the characteristics of the nucleic acid sequences of HEV obtained from the murine rodents and house shrews.

## Results

### Prevalence of HEV in murine rodents and shrews

**Fig. 1:**
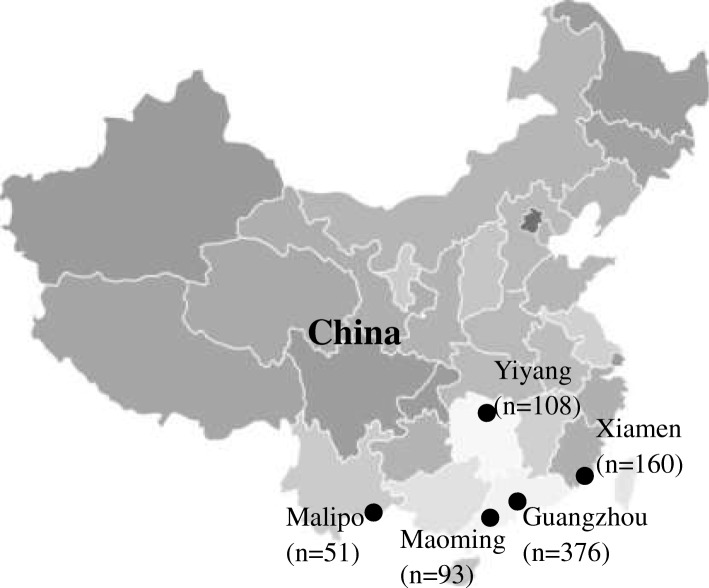
Location of the trapping sites of the murine rodents and house shrews in China. Number of animals trapped are indicated in brackets. Map source: http://image.so.com/v?src=360pic_normal&z=1&i=0&cmg=9923e970972c38717e105d62081f0181&q=%E4%B8%AD%E5%9B%BD%E5%9C%B0%E5%9B%BE&correct=%E4%B8%AD%E5%9B%BD%E5%9C%B0%E5%9B%BE&cmsid=4372c66d63a3bc1a0c3a77413cee395a&cmran=0&cmras=0&cn=0&gn=0&kn=11#multiple=0&gsrc=1&dataindex=44&id=8c7daffc8205d4592f8e4a6e8c0e25e2&currsn=0&jdx=44&fsn=71

From 2014 to 2017, a total of 788 animals were trapped. Liver and fecal specimens were collected from 456 *R. norvegicus*, 64 *Rattus tanezumi* (*R. tanezumi*), 93 *Rattus losea* (*R. losea*), 1 *Bandicota indica* (*B. indica*), and 174 *Suncus murinus* (*S. murinus*) (Fig. 1 and Table 1).

**Table 1:** Prevalence of HEV in liver and fecal samples (%, n)

Sample	Family	Species	Guangzhou	Yiyang	Xiamen	Malipo	Maoming	Total
Liver	*Muridae*	*Rattus norvegicus*	14.57 (29/199)	30.34 (27/89)	12.50 (4/32)	14.00 (7/50)	29.07 (25/86)	20.18 (92/456)
		*Rattus tanezumi*	0 (0/13)	10.53 (2/19)	16.67 (4/24)	0 (0/1)	0 (0/7)	9.48 (6/64)
		*Rattus losea*	-	-	27.96 (26/93)	-	-	27.96 (26/93)
		*Bandicota indica*	-	-	0 (0/1)	-	-	0 (0/1)
		Subtotal	13.68 (29/212)	26.85 (29/108)	22.67 (34/150)	13.72 (7/51)	26.88 (25/93)	20.19 (124/614)
	*Soricidae*	*Suncus murinus*	0 (0/164)	-	10.00 (1/10)	-	-	0.57 (1/174)
	Total		7.71 (29/376)	26.85 (29/108)	21.88 (35/160)	13.72 (7/51)	26.88 (25/93)	15.86 (125/788)
Fecal	*Muridae*	*Rattus norvegicus*	42.21 (84/199)	65.17 (58/89)	28.12 (9/32)	66.00 (33/50)	59.30 (51/86)	51.54 (235/456)
		*Rattus tanezumi*	15.38 (2/13)	68.42 (13/19)	12.50 (3/24)	100 (1/1)	28.57 (2/7)	32.81 (21/64)
		*Rattus losea*	-	-	26.88 (25/93)	-	-	26.88 (25/93)
		*Bandicota indica*	-	-	0 (0/1)	-	-	0 (0/1)
		Subtotal	40.57 (86/212)	65.74 (71/108)	24.66 (37/150)	66.67 (34/51)	56.99 (53/93)	45.76 (281/614)
	*Soricidae*	*Suncus murinus*	3.05 (5/164)	-	40.00 (4/10)	-	-	5.17 (9/174)
	Total		24.20 (91/376)	65.74 (71/108)	25.62 (41/160)	66.67 (34/51)	56.99 (53/93)	36.80 (290/788)

According to nested broad-spectrum RT-PCR and nested PCR, 20.19% (124/614) of the liver tissue samples and 45.76% (281/614) of the fecal samples from murine rodents were positive for HEV. Of the 281 fecal-HEV-positive murine rodents, 84 (29.89%) also tested positive for the virus in their liver tissue. Nine (5.17%) fecal samples from house shrews were positive for HEV. Among these nine fecal-HEV-positive house shrews, one of them also was positive for HEV in the liver (Table 1).

Among the five regions of sampling, the prevalence of HEV in liver tissue samples ranged from 7.71–26.88%, while that in fecal samples ranged from 24.20–66.67%. Detection rates of HEV in liver samples from different species of animals ranged from 0 to 27.96%. With regard to fecal samples, the positive rates of HEV in different species of animals ranged from 0 to 51.54%. The positive rate of HEV in liver tissue samples from *R. losea* was significantly higher than in other species (χ^2^ = 4.399, *P* < 0.05). For fecal samples, the positive rate for HEV was significantly higher in *R. norvegicus* than in other species (χ^2^ = 86.309, *P* < 0.001) (Table 1).

### Phylogenetic analysis

Partial ORF1 sequences and ORF1–ORF2 sequences were detected from liver tissue samples and fecal samples.

The identity among the representative partial ORF1 sequences obtained in our study ranged from 77.2–100% (Additional file 1: Table S1). Among these sequences, two sequences detected in *R. losea* from Xiamen City were totally identical (XM16 and XM27). Additionally, one sequence detected in house shrew from Xiamen City was identical to another sequence detected in *R. norvegicus* from Yunnan Province (XM54 and MLP51). The identity between the representative sequences obtained in this study and that reported in a previous study were also compared (GQ504010) [8] (Table 2). HEV detected from *R. losea* had a lower percentage of identity than that detected from other species. In this region, the percentage of identity at the amino acid level is greater than the percentage of identity at the nucleotide level.

**Table 2:** Nucleotide (nt) and amino acid sequence identity for the region spanning nt positions 4139–4393 or amino acid positions 1379–1462, based on strain GQ504010

Species	Nt identity (%)	Amino acid identity (%)
*Rattus norvegicus* (13^a^)	81.5–86.6	88.2–96.4
*Rattus tanezumi* (2^a^)	84.3–86.2	95.2
*Rattus losea* (3^a^)	78.4–79.6	94.1
*Suncus murinus* (1^a^)	83.9	95.2

^a^number of the sequences

GQ504010, GenBank accession number of a HEV strain isolated from *R. norvegicus* from Germany

Phylogenetic trees constructed by using two different methods were similar. Here, we only showed the phylogenetic trees constructed with MrBayes (version 3.2) (Figs. 2, 3, 4, 5, 6, 7 and 8). The phylogenetic trees obtained based on the neighbor-joining method are shown in the supplementary information file (Additional file 1: Figures S1-S7).

**Fig. 2:**
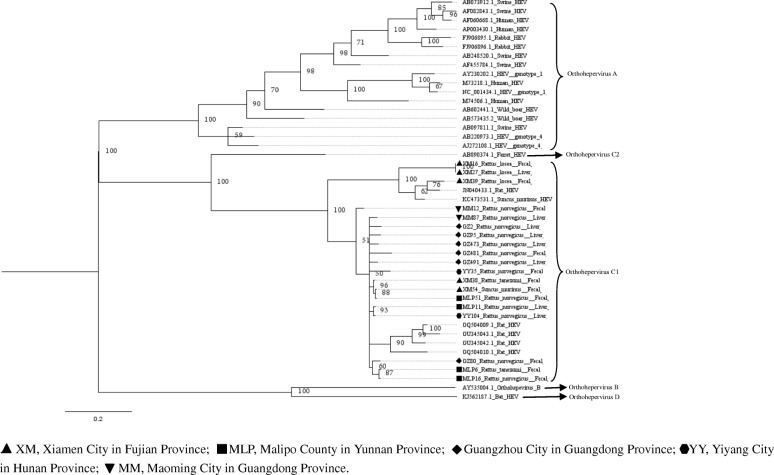
Phylogenetic tree constructed based on partial nucleotide sequences of partial ORF1 regions (255-nt) of 19 HEV strains (MrBayes, GTR + G + I nucleotide substitution model). Twenty five representative HEV isolates derived from rats, swine, humans, rabbits, wild boars, bat, and ferret are included for comparison. Percentages of the posterior probability (PP) values are indicated

**Fig. 3:**
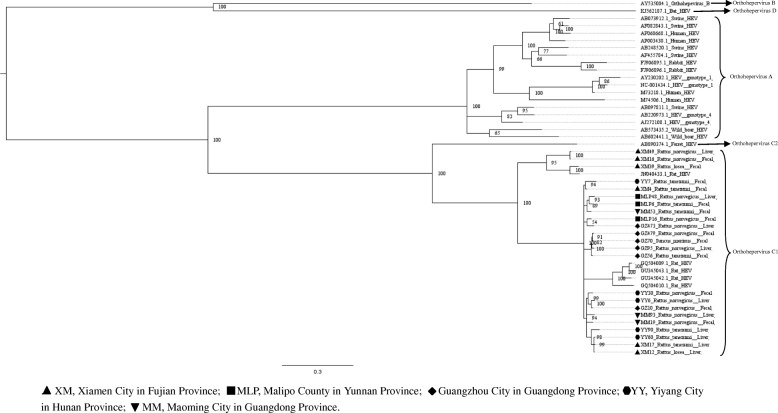
Phylogenetic tree constructed based on partial nucleotide sequences of partial ORF1–ORF2 regions (769-nt) of 23 HEV isolates (MrBayes, GTR + G + I nucleotide substitution model). Five rat HEV isolates and 20 HEV isolates derived from swine, humans, rabbits, wild boars, bat, and ferret are included for comparison. Percentages of the PP values are indicated

**Fig. 4:**
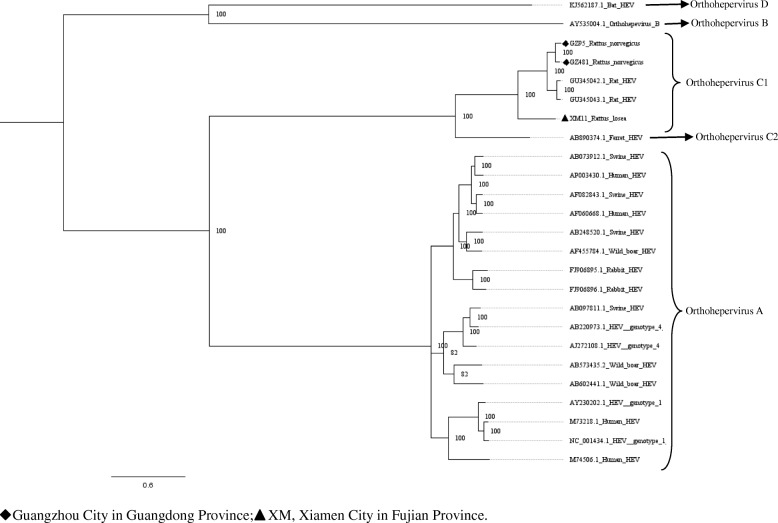
Phylogenetic tree constructed based on near full-length genomes of HEV (MrBayes, GTR + G + I nucleotide substitution model). Three rat HEV isolates and 22 HEV isolates derived from swine, humans, rabbits, wild boars, bat, and ferret are included for comparison. Percentages of the PP values are indicated

**Fig. 5:**
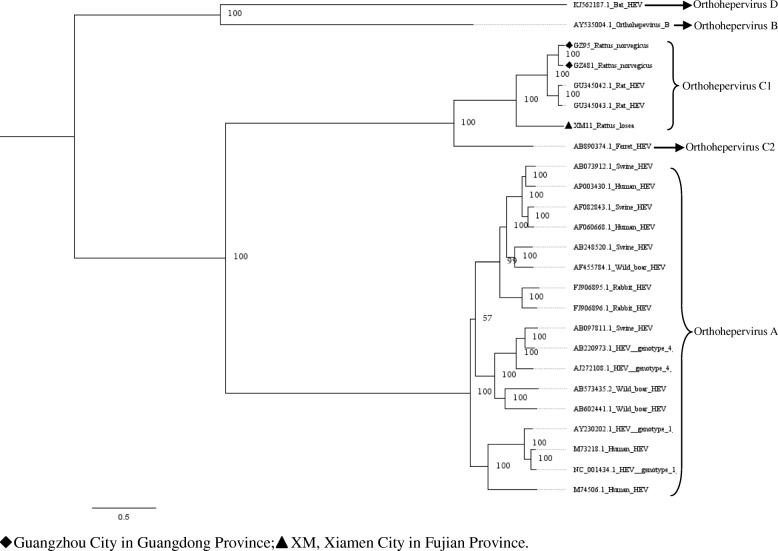
Phylogenetic tree constructed based on partial ORF1 of the near full-length genomes of HEV (MrBayes, GTR + G + I nucleotide substitution model). Three rat HEV isolates and 22 HEV isolates derived from swine, humans, rabbits, wild boars, bat, and ferret are included for comparison. Percentages of the PP values are indicated

**Fig. 6:**
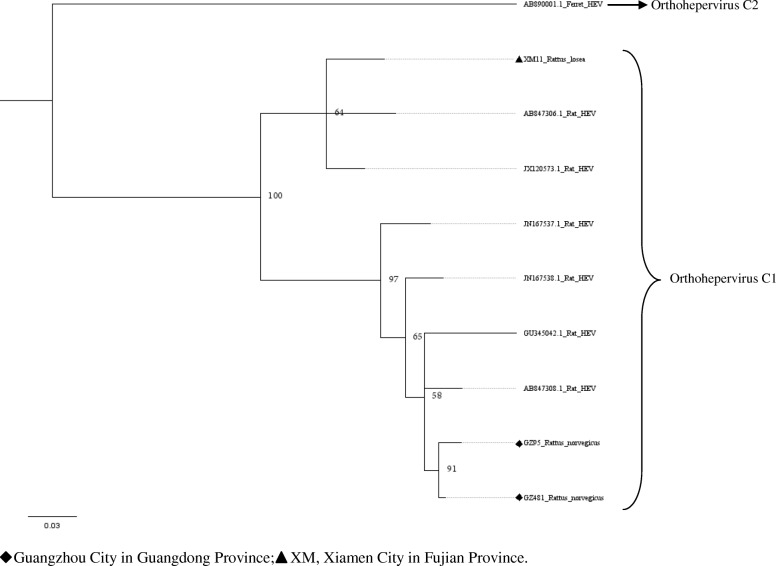
Phylogenetic tree constructed based on partial ORF4 of the near full-length genomes of HEV (MrBayes, GTR + G + I nucleotide substitution model). Three rat HEV isolates and seven HEV isolates derived from rats and ferret are included for comparison. Percentages of the PP values are indicated

**Fig. 7:**
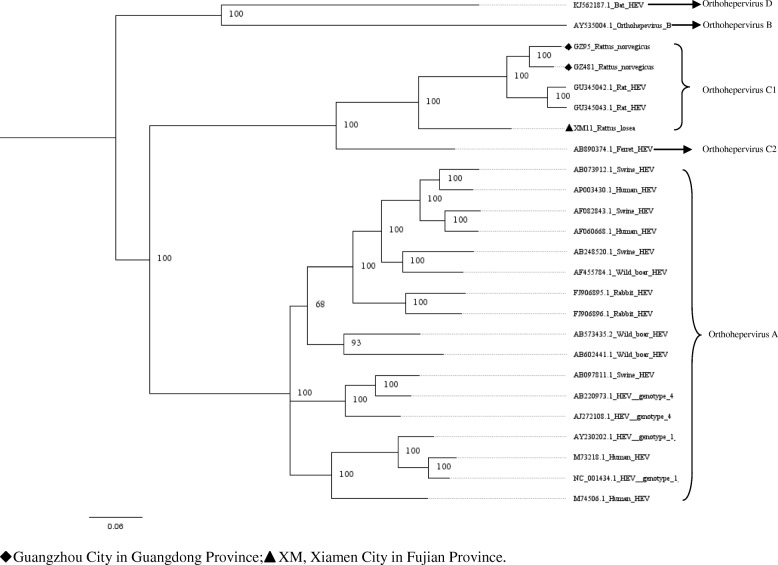
Phylogenetic tree constructed based on partial ORF2 of the near full-length genomes of HEV (MrBayes, GTR + G + I nucleotide substitution model). Three rat HEV isolates and 22 HEV isolates derived from swine, humans, rabbits, wild boars, bat, and ferret are included for comparison. Percentages of the PP values are indicated

**Fig. 8:**
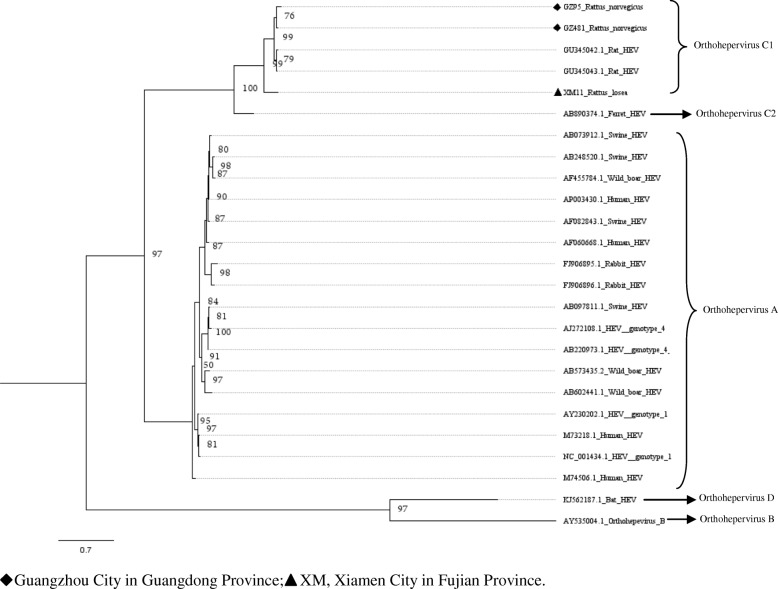
Phylogenetic tree constructed based on ORF3 of the near full-length genomes of HEV (MrBayes, GTR + G + I nucleotide substitution model). Three rat HEV isolates and 22 HEV isolates derived from swine, humans, rabbits, wild boars, bat, and ferret are included for comparison. Percentages of the PP values are indicated

Phylogenetic trees were generated for the region spanning the nucleotides (nt) 4139 to 4393 (numbering based on a HEV sequence from *R. norvegicus*, GenBank accession number: JN167538), with the selected representative fragment sequences from our study and nucleotide sequences of HEV from GenBank (Fig. 2). From the result of the phylogenetic analysis, all of the novel sequences were found to belong to the species *Orthohepevirus* C1. The HEV sequence detected in house shrew clustered with rat HEV sequences from murine rodents, indicating that shrew HEV was similar to rat HEV. Excluding three sequences detected in *R. losea* from Xiamen City, all selected sequences were clustered with the HEV strains detected in Germany.

Upon analyzing the representative nested PCR products of rat HEV, the similarity ranged from 77.1–99.6% (Additional file 1: Table S2). As compared with the sequence reported in a previous study (GQ504010) [8], the HEV sequences detected in *R. losea* have a lower degree of identity than the sequences detected in other species (Table 3). The amino acid sequences of this region also have a higher percentage of identity than the nucleotide sequences, similar to our findings in the analysis of the partial ORF1 sequences. Phylogenetic tree was generated for the region spanning the nt 4157 to 4925 (numbering based on HEV sequence from *R. norvegicus*, GenBank accession number: JN167538), with the representative fragment sequences from this study and HEV sequences from GenBank. Phylogenetic analysis showed that all of the sequences belonged to the species *Orthohepevirus* C1. Two sequences from *R. norvegicus* and one sequence from *R. losea* clustered together with a rat HEV sequence from Asia, while the other HEV strains clustered with rat sequences from Europe (Fig. 3).

**Table 3:** Nucleotide (nt) and amino acid sequence identity for the region spanning nt positions 4157–4925 or amino acid positions 1385–1636, based on strain GQ504010

Species	Nt identity (%)	Amino acid identity (%)
*Rattus norvegicus* (12^a^)	77.7–85.3	93.3–97.2
*Rattus tanezumi* (8^a^)	84.2–85.1	96.4–96.8
*Rattus losea* (2^a^)	77.5–84.5	93.7–96.4
*Suncus murinus* (1^a^)	84.9	95.6

^a^number of the sequences

GQ504010, GenBank accession number of a HEV strain isolated from *R. norvegicus* from Germany

### Characterization of the HEV genome

**Fig. 9:**
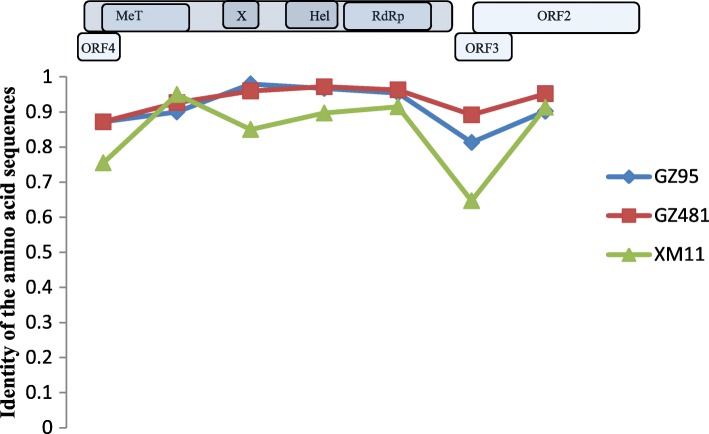
The identity of the different regions of the amino acid sequences (XM11, GZ95, and GZ481), based on rat HEV (GenBank accession no. GU345042)

In murine rodents, three near full-length HEV genomes (GenBank accession numbers: MH729810–MH729812) detected in *R. norvegicus* (GZ95 and GZ481) and *R. losea* (XM11) were obtained. The length of these sequences ranged between the nt 6692 and 6790. These sequences were compared with the rat HEV sequence reported in a previous study (GU345042) [22]. The XM11 nucleotide and amino acid sequences had a lower percentage of identity than other HEV sequences. Additionally, the identity of the amino acid sequences of three near full-length HEV genomes in the ORF3 region were lower than other regions (Table 4 and Fig. 9). Phylogenetic trees were constructed based on these near full-length sequences and some representative HEV sequences from humans and other species of animals (Figs. 4, 5, 6, 7 and 8). The results showed that the near full-length HEV genomes obtained in this study clustered with rat HEV strains (Fig. 4). Two sequences from *R. norvegicus* in Guangzhou city clustered together in one branch, while the other HEV sequence from *R. losea* was located in another branch of the phylogenetic trees (Figs. 4, 5, 6, 7 and 8).

**Table 4:** Nucleotide (nt) and amino acid identity for different regions of the near-full-length HEV genomes, based on strain GU345042

	Nt identity				Amino acid identity			
	Partial ORF1	Partial OFR2	ORF3	Partial OFR4	Partial ORF1	Partial OFR2	ORF3	Partial OFR4
GZ95	0.836	0.833	0.899	0.945	0.907	0.902	0.813	0.872
GZ481	0.846	0.884	0.935	0.941	0.919	0.952	0.892	0.872
XM11	0.744	0.783	0.805	0.893	0.864	0.913	0.647	0.755

GU345042, GenBank accession number of a HEV strain isolated from *R. norvegicus* from Germany

Five partial HEV nucleic acid sequences from house shrews, whose length ranged from 778 bp to 825 bp, were obtained (GenBank accession numbers: MH729813–MH729817). The selected representative house shrew sequence was clustered with rat HEV sequences (Figs. 2 and 3).

## Discussion

In this study, we investigated HEV in four species of murine rodents and house shrews from four provinces in China via nested broad-spectrum RT-PCR assay and nested PCR method for rat HEV [8, 23]. During the conduct of this study, care was taken to limit contamination and a strict operating standard was followed during each experimental step, with a negative control established for each experiment. Our study evaluated the geographical distribution of HEV in murine rodents and house shrews in China.

HEV-positivity was detected in all of the cities investigated in this study, indicating that HEV is widely distributed among the three common species of murine rodents (*R. norvegicus*, *R. tanezumi*, and *R. losea*) in China. Specifically, 20.19% (124/614) of the liver tissue samples and 45.76% (281/614) of the fecal samples from the murine rodents were positive for HEV. In comparison, HEV-positivity in *R. norvegicus* was found to be only 2.87% in a previous study conducted in China [16]. The reason for the discrepancy in the detection rates of HEV in the present study versus the previous study is unclear. One explanation may be variations in the PCR methods used, which may affect the results obtained. The primers of two PCR methods we selected were designed based on the well-conserved regions of all types of HEV and the rat HEV genome, which may have contributed to the high detection rates of HEV in this study.

Positive liver samples were detected in all of the murine rodents, except *B. indica*. The positive liver samples indicated that HEV was capable of infecting these animals and subsequently replicating in them. The high prevalence and effective replication of HEV furthermore suggested that these animals are hosts of HEV.

In our study, the detection rate of HEV in house shrews was lower than in other species. This result was similar to that of the previous study conducted in China, in which only one of the 196 house shrews was positive for HEV [16]. Another study conducted in Nepal also reported the low prevalence of anti-HEV Ig-G in house shrews [24]. These indicate that house shrews might not be a natural host of HEV.

HEV has been detected in several species of murine rodents, such as *R. norvegicus*, *R. flavipectus*, and *R. losea*. In our study, the detection rate for HEV in *R. losea* in liver samples was higher than in other species of animals; this result was consistent with the findings of a previous study, indicating that *R. losea* is an important natural host for HEV [25]. *R. norvegicus* had the highest prevalence of HEV in fecal samples (Table 1). This result might be explained by the rats’ habitats. *R. norvegicus* mainly inhabits sewers and garbage dumps. Poor sanitation is conducive to the spread of HEV, which might explain the high HEV detection rate in the fecal samples from *R. norvegicus*. Further study is needed to explain why the detection rate in the liver samples from *R. losea* was higher than that in *R. norvegicus*, while the positive rate in fecal samples from *R. losea* was lower than that in *R. norvegicus.*

To our knowledge, we report the first near full-length of HEV genome from *R. losea* in our study. We also present the first near full-length rat HEV genomes in *R. norvegicus* from China. According to the phylogenetic analysis, all of the HEV sequences detected in the trapped murine rodents belonged to rat HEV. This result was in agreement with those of most previous studies [16, 26, 27], indicating that murine rodents are exclusively susceptible to rat HEV. However, some previous studies reported that HEV genotype 3 was detected in *R. norvegicus* [18]. Follow-up studies are necessary to confirm whether other genotypes of HEV can infect murine rodents.

Near full-length genome analysis showed that rat HEV sequences obtained in our study clustered with other rat HEV strains that were reported previously (Fig. 4). When analyzing the identity of the different regions between amino acid sequences, the partial ORF2 region seems to be a relatively conserved region (Fig. 9). The ORF2 region is known to be associated with capsid assembly and has many immune epitopes; thus, the ORF2 protein can induce strong immune responses [28]. In recent years, anti-rat HEV antibodies have been detected in forest workers and febrile patients [17, 29]. This suggests that rat HEV might infect humans and lead to liver diseases. However, further research is necessary to investigate the transmission of HEV between humans and rats.

Partial ORF1 and the ORF1–ORF2 sequences obtained in animals from different regions were clustered together, and we found that two sequences detected in *R. losea* from Xiamen City were totally identical, indicating that the HEV infecting the same species of animals were similar (Figs. 2 and 3 and Additional file 1: Table S1). In our study, HEV detected in *R. losea* showed a lower percentage of identity versus in other animal species (Tables 2 and 3). When comparing the three near full-length HEV sequences obtained in our study with the rat HEV sequence reported in a previous study (GU345042) [8], the near-full-length sequence from *R. losea* in Xiamen City also showed a lower percentage of identity than the sequences from *R. norvegicus* in Guangzhou City (Table 4). The result of the phylogenetic analysis showed that two sequences from *R. norvegicus* in Guangzhou City clustered together, while the other HEV strains from *R. losea* were in another branch of the phylogenetic trees (Figs. 5, 6, 7 and 8). This might be due to the difference in HEV infection as a result of host-specificity.

The percentages of identity at the amino acid level in the partial ORF1 and ORF1-ORF2 regions were greater than the percentages of identity at the nucleotide level (Tables 2 and 3). This might be explained by the presence of degenerate codons.

A 281 bp fragment sequence of HEV from house shrews was reported in a previous study [30]. In this investigation, 778 bp to 825 bp partial nucleic acid sequences of HEV from house shrews were obtained. One partial ORF1 sequence from house shrew obtained in our study was identical to another sequence detected in *R. norvegicus*. The result of phylogenetic analysis based on partial sequences of ORF1 region showed that HEV sequence detected in house shrew clustered with rat HEV sequences instead of the HEV sequence detected from house shrew reported in previous studies (Fig. 2). This may be explained by the interspecies transmission or spillover infection of HEV between murine rodents and house shrews [30].

## Conclusions

There was a high prevalence of HEV in fecal samples of *R. norvegicus*, while a high prevalence of HEV was observed in the liver samples of *R. losea*. Murine rodents and house shrews shared a high identity genome of HEV, suggesting that HEV might cross transmit among different animal species.

## Methods

### Samples

Between 2014 and 2017, rodents and shrews were captured near human residences with cage traps. These animals were captured in five regions of four provinces in China, as follows: Yiyang City in Hunan Province, Xiamen City in Fujian Province, Maoming City and Guangzhou City in Guangdong Province, and Malipo County in Yunan Province (Fig. 1). Inhalational anesthesia was performed with diethyl ether, and the dosage of diethyl ether was adjusted according to the heart rate, respiratory frequency, corneal reflection and extremity muscle tension of the animal. Following anesthesia, the rodents and shrews were executed via cervical dislocation by trained personnel. Liver tissue samples were collected by intraperitoneal surgery in the laboratory and stored in RNAlater (Invitrogen, California, United States). The fecal samples were soaked in phosphate-buffered saline (PBS). The liver tissue and fecal samples were stored at − 80 °C and thawed at 4 °C prior to processing. The species of the trapped animals were identified by morphological identification and sequencing of the cytochrome B (*cytB*) gene [31].

### Extraction of nucleic acid and detection of HEV

Total RNA and DNA were extracted from ~ 20 mg of liver tissue samples or 200 μL aliquot of fecal samples by using the MiniBEST Viral RNA/DNA Extraction Kit (TaKaRa, Kusatsu, Japan). We simultaneously used two polymerase chain reaction (PCR) methods for detecting HEV, one of which was a nested broad-spectrum PCR to amplify a 334 bp ORF1 fragment of all known HEV strains [8], while the other was a nested PCR to amplify the ORF1–ORF2 region of rat HEV [23]. The amplified products were separated on a 1.5% agarose gel, and the positive samples were sent to the Beijing Genomics Institute (Shenzhen, China) for sequencing.

### Genome sequencing

Based on six HEV genome sequences from GenBank (GenBank accession numbers: JX120573, AB847307, GU345042, JN167538, KM516906, and LC225389), we designed eight pairs of primers to amplify the near full-length genomes of HEV (Table 5). After sequencing all of the fragments, the Lasergene SeqMan software (DNASTAR; Madison, WI, USA) was used to assemble the sequences.

**Table 5:** HEV near-full-length PCR primers

Primer	Orientation	Sequence (5'-3')	Target fragment (base pairs)
HEV-F15	Sense (first/second round)	AGACCCATCARTATGTCG	921 (this study)
HEV-R935	Antisense (second round)	GTDGAYCKRGMCTTCTCACA	
HEV-R977	Antisense (first round)	CATRAGYCKRTCCCADAT	
HEV-F827	Sense (first/second round)	CATCTATGTGCGCAGCCTGT	1340 (this study)
HEV-R2166	Antisense (second round)	GTGGACAAACTGGGTGCGATC	
HEV-R2228	Antisense (first round)	GTGCCACAGCGTGTATTATAG	
HEV-F2084	Sense (first/second round)	GCNGTNTATGARGGRGAY	955 (this study)
HEV-R3038	Antisense (second round)	CAAAATCWATNGCNGGGATCTG	
HEV-R3053	Antisense (first round)	ATVAGVCCCTTGCTYTCAAAATC	
HEV-F2868	Sense (first/second round)	TKAARGCNCARTGGMGDG	841 (this study)
HEV-R3708	Antisense (second round)	ARBAYDGTCACCTGVTCHC	
HEV-R3740	Antisense (first round)	ATRCGRCARTGCACDGT	
HEV-F3539	Sense (first/second round)	ACCAACTTGCAGGATATAG	699 (this study)
HEV-R4237	Antisense (second round)	AAACTCGCTAAAATCATTCTCAA	
HEV-R4253	Antisense (first round)	ATTCTGGGTGCTGTCAAACTCG	
HEV-F4980	Sense (first/second round)	TCGTGCTCGTGYTTTTGCT	1029 (this study)
HEV-R6008	Antisense (second round)	CCTATRTCRCCYACMCCRTT	
HEV-R6014	Antisense (first round)	CCCTTRCCTATRTCRCCYAC	
HEV-F5552	Sense (first/second round)	GTRTCAATGTCRTTYTGG	1153 (this study)
HEV-R6704	Antisense (second round)	RTTAACAGGYCCAGYACC	
HEV-R6836	Antisense (first round)	ATWGCATCAGCMACGAGGCA	
HEV-F6300	Sense (first/second round)	CAACTGGCGGTCTGGTGATGTC	535 (this study)
HEV-R6881	Antisense (second round)	AGACACTGTCGGCTGCTGC	
HEV-R6834	Antisense (first round)	GCATCAGCCACGAGGCAGG	
HE607	Sense (first round)	CTTGGTTYAGGGCCATAGAG	880 [23]
HE604	Antisense (first round)	CAGCAGCGGCACGAACAGCA	
HE608	Sense (second round)	TTYAGGGCCATAGAGAAGGC	
HE606	Antisense (second round)	ACAGCAAAAGCACGAGCACG	

### Phylogenetic analysis

The selected representative ORF1 fragments and ORF1–ORF2 fragments and all of the near full-length sequences obtained in this study were aligned with HEV sequences from humans and different species of animals obtained via GenBank by using the ClustalW multiple sequence alignment program in MEGA (version 7.0; Oxford Molecular Ltd., Cambridge, UK), respectively. The phylogenetic trees were constructed based on the ORF1 fragments, ORF1–ORF2 fragments, and the near full-length sequences. We constructed the phylogenetic trees via MrBayes (version 3.2) using a GTR + G + I nucleotide substitution matrix; two million Markov chain Monte Carlo (MCMC) iterations were sampled every 100 steps in order to obtain 20,000 trees, and burn-in was generally 25% of tree replicates [32, 33]. The phylogenetic trees were also constructed based on the neighbor-joining method in MEGA, with 1000 bootstrap replicates.

The identity of the different regions of the amino acid sequences and nucleotide sequences were estimated by use of the Sequence Identity Matrix program in BioEdit (version 7.2.5).

### Statistical analysis

Data were analyzed using the Statistical Product and Service Solutions software (SPSS, version 13.0; IBM Corp., Armonk, NY, USA). The positive rates of HEV among different species of animals were analyzed using chi-square tests. A *p*-value of 0.05 was considered to be statistically significant.

### Ethics guidelines

The protocol of this study was approved by the Ethics Committee of the Institutional Animal Care and Use Committee of Southern Medical University in Guangzhou, China.

## Additional files

Additional file 1: **Table S1.** Identity of the representative nucleotide (nt) sequences (nt positions 4139–4393) obtained in this study. **Table S2.** Identity of the representative nucleotide (nt) sequences (nt positions 4157–4925) obtained in this study. **Figure S1.** Phylogenetic tree constructed by the neighbor-joining method based on partial nucleotide sequences of ORF1 regions (255 nt) of 19 HEV strains. Twenty five representative HEV isolates derived from rats, swine, humans, rabbits, wild boars, bat and ferret are included for comparison. Bootstrap support of branches (1000 replication) is indicated. **Figure S2.** Phylogenetic tree constructed by the neighbor-joining method based on partial nucleotide sequences of ORF1 -ORF2 regions (769 nt) of 23 HEV isolates. Five rat HEV isolates and 20 HEV isolates derived from swine, humans, rabbits, wild boars, bat and ferret are included for comparison. Bootstrap support of branches (1000 replication) is indicated. **Figure S3.** Phylogenetic tree constructed by the neighbor-joining method based on near full-length genomes of HEV. Three rat HEV isolates and 22 HEV isolates derived from swine, humans, rabbits, wild boars, bat and ferret are included for comparison. Bootstrap support of branches (1000 replication) is indicated. **Figure S4.** Phylogenetic tree constructed by the neighbor-joining method based on ORF1 of the near full-length genomes of HEV. Three rat HEV isolates and 22 HEV isolates derived from swine, humans, rabbits, wild boars, bat and ferret are included for comparison. Bootstrap support of branches (1000 replication) is indicated. **Figure S5.** Phylogenetic tree constructed by the neighbor-joining method based on ORF4 of the near full-length genomes of HEV. Three rat HEV isolates and 7 HEV isolates derived from rats and ferret are included for comparison. Bootstrap support of branches (1000 replication) is indicated. **Figure S6.** Phylogenetic tree constructed by the neighbor-joining method based on ORF2 of the near full-length genomes of HEV. Three rat HEV isolates and 22 HEV isolates derived from swine, humans, rabbits, wild boars, bat and ferret are included for comparison. Bootstrap support of branches (1000 replication) is indicated. **Figure S7.** Phylogenetic tree constructed by the neighbor-joining method based on ORF3 of the near full-length genomes of HEV. Three rat HEV isolates and 22 HEV isolates derived from swine, humans, rabbits, wild boars, bat and ferret are included for comparison. Bootstrap support of branches (1000 replication) is indicated. (DOCX 309 kb)

## Abbreviation

HEV: Hepatitis E virus
