# Supplementary material for: The prevalence and genomic characteristics of hepatitis E virus in murine rodents and house shrews from several regions in China
Source: BMC Vet Res. 2018 Dec 22;14:414. doi: 10.1186/s12917-018-1746-z (PMC6303920; doi:10.1186/s12917-018-1746-z)
Supplement: Supplementary file 1 — Table S1. Identity of the representative nucleotide (nt) sequences (nt positions 4139–4393) obtained in this study. Table S2. Identity of the representative nucleotide (nt) sequences (nt positions 4157–4925) obtained in this study. Figure S1. Phylogenetic tree constructed by the neighbor-joining method based on partial nucleotide sequences of ORF1 regions (255 nt) of 19 HEV strains. Twenty five representative HEV isolates derived from rats, swine, humans, rabbits, wild boars, bat and ferret are included for comparison. Bootstrap support of branches (1000 replication) is indicated. Figure S2. Phylogenetic tree constructed by the neighbor-joining method based on partial nucleotide sequences of ORF1 -ORF2 regions (769 nt) of 23 HEV isolates. Five rat HEV isolates and 20 HEV isolates derived from swine, humans, rabbits, wild boars, bat and ferret are included for comparison. Bootstrap support of branches (1000 replication) is indicated. Figure S3. Phylogenetic tree constructed by the neighbor-joining method based on near full-length genomes of HEV. Three rat HEV isolates and 22 HEV isolates derived from swine, humans, rabbits, wild boars, bat and ferret are included for comparison. Bootstrap support of branches (1000 replication) is indicated. Figure S4. Phylogenetic tree constructed by the neighbor-joining method based on ORF1 of the near full-length genomes of HEV. Three rat HEV isolates and 22 HEV isolates derived from swine, humans, rabbits, wild boars, bat and ferret are included for comparison. Bootstrap support of branches (1000 replication) is indicated. Figure S5. Phylogenetic tree constructed by the neighbor-joining method based on ORF4 of the near full-length genomes of HEV. Three rat HEV isolates and 7 HEV isolates derived from rats and ferret are included for comparison. Bootstrap support of branches (1000 replication) is indicated. Figure S6. Phylogenetic tree constructed by the neighbor-joining method based on ORF2 of the near full-length genomes of HEV. [file 12917_2018_1746_MOESM1_ESM.docx]

Table S1. Identity of the representative nucleotide (nt) sequences (nt positions 4139 - 4393) obtained in this study.

|  | MM87 | MLP11 | GZ2 | GZ80 | GZ95 | MM12 | GZ473 | GZ481 | GZ491 | XM16 | XM27 | XM38 | XM39 | XM54 | MLP6 | MLP16 | MLP51 | YY35 | YY104 |
| --- | --- | --- | --- | --- | --- | --- | --- | --- | --- | --- | --- | --- | --- | --- | --- | --- | --- | --- | --- |
| MM87 | ID | 0.96 | 0.941 | 0.925 | 0.941 | 0.925 | 0.921 | 0.917 | 0.925 | 0.803 | 0.803 | 0.964 | 0.803 | 0.96 | 0.937 | 0.941 | 0.96 | 0.913 | 0.96 |
| MLP11 | 0.96 | ID | 0.949 | 0.933 | 0.933 | 0.925 | 0.929 | 0.917 | 0.933 | 0.803 | 0.803 | 0.972 | 0.8 | 0.976 | 0.945 | 0.941 | 0.976 | 0.921 | 0.992 |
| GZ2 | 0.941 | 0.949 | ID | 0.921 | 0.921 | 0.913 | 0.909 | 0.898 | 0.909 | 0.8 | 0.8 | 0.956 | 0.8 | 0.952 | 0.917 | 0.921 | 0.952 | 0.898 | 0.949 |
| GZ80 | 0.925 | 0.933 | 0.921 | ID | 0.937 | 0.913 | 0.941 | 0.905 | 0.945 | 0.78 | 0.78 | 0.937 | 0.784 | 0.933 | 0.956 | 0.96 | 0.933 | 0.909 | 0.933 |
| GZ95 | 0.941 | 0.933 | 0.921 | 0.937 | ID | 0.929 | 0.933 | 0.89 | 0.929 | 0.819 | 0.819 | 0.937 | 0.811 | 0.933 | 0.933 | 0.937 | 0.933 | 0.917 | 0.933 |
| MM12 | 0.925 | 0.925 | 0.913 | 0.913 | 0.929 | ID | 0.925 | 0.898 | 0.905 | 0.811 | 0.811 | 0.933 | 0.811 | 0.929 | 0.917 | 0.921 | 0.929 | 0.913 | 0.933 |
| GZ473 | 0.921 | 0.929 | 0.909 | 0.941 | 0.933 | 0.925 | ID | 0.901 | 0.941 | 0.811 | 0.811 | 0.941 | 0.803 | 0.937 | 0.945 | 0.949 | 0.937 | 0.921 | 0.937 |
| GZ481 | 0.917 | 0.917 | 0.898 | 0.905 | 0.89 | 0.898 | 0.901 | ID | 0.909 | 0.772 | 0.772 | 0.921 | 0.792 | 0.917 | 0.917 | 0.921 | 0.917 | 0.878 | 0.917 |
| GZ491 | 0.925 | 0.933 | 0.909 | 0.945 | 0.929 | 0.905 | 0.941 | 0.909 | ID | 0.807 | 0.807 | 0.929 | 0.803 | 0.933 | 0.949 | 0.945 | 0.933 | 0.917 | 0.925 |
| XM16 | 0.803 | 0.803 | 0.8 | 0.78 | 0.819 | 0.811 | 0.811 | 0.772 | 0.807 | ID | 1 | 0.807 | 0.831 | 0.803 | 0.784 | 0.788 | 0.803 | 0.796 | 0.803 |
| XM27 | 0.803 | 0.803 | 0.8 | 0.78 | 0.819 | 0.811 | 0.811 | 0.772 | 0.807 | 1 | ID | 0.807 | 0.831 | 0.803 | 0.784 | 0.788 | 0.803 | 0.796 | 0.803 |
| XM38 | 0.964 | 0.972 | 0.956 | 0.937 | 0.937 | 0.933 | 0.941 | 0.921 | 0.929 | 0.807 | 0.807 | ID | 0.803 | 0.996 | 0.933 | 0.937 | 0.996 | 0.925 | 0.972 |
| XM39 | 0.803 | 0.8 | 0.8 | 0.784 | 0.811 | 0.811 | 0.803 | 0.792 | 0.803 | 0.831 | 0.831 | 0.803 | ID | 0.8 | 0.8 | 0.803 | 0.8 | 0.76 | 0.8 |
| XM54 | 0.96 | 0.976 | 0.952 | 0.933 | 0.933 | 0.929 | 0.937 | 0.917 | 0.933 | 0.803 | 0.803 | 0.996 | 0.8 | ID | 0.937 | 0.933 | 1 | 0.929 | 0.968 |
| MLP6 | 0.937 | 0.945 | 0.917 | 0.956 | 0.933 | 0.917 | 0.945 | 0.917 | 0.949 | 0.784 | 0.784 | 0.933 | 0.8 | 0.937 | ID | 0.996 | 0.937 | 0.921 | 0.937 |
| MLP16 | 0.941 | 0.941 | 0.921 | 0.96 | 0.937 | 0.921 | 0.949 | 0.921 | 0.945 | 0.788 | 0.788 | 0.937 | 0.803 | 0.933 | 0.996 | ID | 0.933 | 0.917 | 0.941 |
| MLP51 | 0.96 | 0.976 | 0.952 | 0.933 | 0.933 | 0.929 | 0.937 | 0.917 | 0.933 | 0.803 | 0.803 | 0.996 | 0.8 | 1 | 0.937 | 0.933 | ID | 0.929 | 0.968 |
| YY35 | 0.913 | 0.921 | 0.898 | 0.909 | 0.917 | 0.913 | 0.921 | 0.878 | 0.917 | 0.796 | 0.796 | 0.925 | 0.76 | 0.929 | 0.921 | 0.917 | 0.929 | ID | 0.921 |
| YY104 | 0.96 | 0.992 | 0.949 | 0.933 | 0.933 | 0.933 | 0.937 | 0.917 | 0.925 | 0.803 | 0.803 | 0.972 | 0.8 | 0.968 | 0.937 | 0.941 | 0.968 | 0.921 | ID |

Table S2. Identity of the representative nucleotide (nt) sequences (nt positions 4157 - 4925) obtained in this study.

|  | YY90 | YY60 | YY30 | YY7 | YY6 | MLP48 | MLP16 | MLP6 | XM49 | XM39 | XM17 | XM12 | GZ479 | GZ473 | GZ70 | MM93 | MM53 | MM19 | GZ10 | XM16 | XM4 | GZ56 | GZ95 |
| --- | --- | --- | --- | --- | --- | --- | --- | --- | --- | --- | --- | --- | --- | --- | --- | --- | --- | --- | --- | --- | --- | --- | --- |
| YY90 | ID | 0.959 | 0.937 | 0.918 | 0.933 | 0.921 | 0.931 | 0.929 | 0.789 | 0.771 | 0.958 | 0.959 | 0.919 | 0.924 | 0.921 | 0.914 | 0.928 | 0.938 | 0.94 | 0.788 | 0.942 | 0.924 | 0.919 |
| YY60 | 0.959 | ID | 0.949 | 0.928 | 0.942 | 0.931 | 0.937 | 0.934 | 0.791 | 0.775 | 0.993 | 0.994 | 0.925 | 0.923 | 0.928 | 0.925 | 0.938 | 0.947 | 0.949 | 0.79 | 0.949 | 0.931 | 0.925 |
| YY30 | 0.937 | 0.949 | ID | 0.94 | 0.959 | 0.936 | 0.938 | 0.936 | 0.793 | 0.773 | 0.95 | 0.951 | 0.932 | 0.927 | 0.932 | 0.931 | 0.947 | 0.951 | 0.966 | 0.791 | 0.955 | 0.94 | 0.932 |
| YY7 | 0.918 | 0.928 | 0.94 | ID | 0.928 | 0.921 | 0.94 | 0.929 | 0.79 | 0.773 | 0.927 | 0.928 | 0.924 | 0.923 | 0.924 | 0.915 | 0.927 | 0.931 | 0.934 | 0.789 | 0.954 | 0.929 | 0.921 |
| YY6 | 0.933 | 0.942 | 0.959 | 0.928 | ID | 0.924 | 0.937 | 0.934 | 0.784 | 0.782 | 0.944 | 0.945 | 0.92 | 0.928 | 0.923 | 0.927 | 0.944 | 0.947 | 0.983 | 0.782 | 0.949 | 0.928 | 0.923 |
| MLP48 | 0.921 | 0.931 | 0.936 | 0.921 | 0.924 | ID | 0.937 | 0.968 | 0.786 | 0.786 | 0.932 | 0.931 | 0.941 | 0.932 | 0.941 | 0.912 | 0.962 | 0.934 | 0.928 | 0.785 | 0.947 | 0.941 | 0.938 |
| MLP16 | 0.931 | 0.937 | 0.938 | 0.94 | 0.937 | 0.937 | ID | 0.966 | 0.793 | 0.788 | 0.938 | 0.94 | 0.932 | 0.954 | 0.934 | 0.914 | 0.942 | 0.938 | 0.941 | 0.791 | 0.962 | 0.937 | 0.932 |
| MLP6 | 0.929 | 0.934 | 0.936 | 0.929 | 0.934 | 0.968 | 0.966 | ID | 0.784 | 0.786 | 0.938 | 0.937 | 0.933 | 0.94 | 0.933 | 0.918 | 0.964 | 0.938 | 0.936 | 0.782 | 0.958 | 0.936 | 0.931 |
| XM49 | 0.789 | 0.791 | 0.793 | 0.79 | 0.784 | 0.786 | 0.793 | 0.784 | ID | 0.851 | 0.793 | 0.791 | 0.789 | 0.79 | 0.791 | 0.785 | 0.791 | 0.798 | 0.785 | 0.996 | 0.791 | 0.801 | 0.795 |
| XM39 | 0.771 | 0.775 | 0.773 | 0.773 | 0.782 | 0.786 | 0.788 | 0.786 | 0.851 | ID | 0.776 | 0.775 | 0.778 | 0.785 | 0.778 | 0.769 | 0.789 | 0.782 | 0.78 | 0.853 | 0.775 | 0.788 | 0.782 |
| XM17 | 0.958 | 0.993 | 0.95 | 0.927 | 0.944 | 0.932 | 0.938 | 0.938 | 0.793 | 0.776 | ID | 0.996 | 0.927 | 0.921 | 0.929 | 0.927 | 0.942 | 0.949 | 0.95 | 0.791 | 0.95 | 0.932 | 0.927 |
| XM12 | 0.959 | 0.994 | 0.951 | 0.928 | 0.945 | 0.931 | 0.94 | 0.937 | 0.791 | 0.775 | 0.996 | ID | 0.925 | 0.923 | 0.928 | 0.928 | 0.941 | 0.95 | 0.951 | 0.79 | 0.951 | 0.931 | 0.925 |
| GZ479 | 0.919 | 0.925 | 0.932 | 0.924 | 0.92 | 0.941 | 0.932 | 0.933 | 0.789 | 0.778 | 0.927 | 0.925 | ID | 0.924 | 0.989 | 0.91 | 0.936 | 0.931 | 0.927 | 0.788 | 0.945 | 0.984 | 0.984 |
| GZ473 | 0.924 | 0.923 | 0.927 | 0.923 | 0.928 | 0.932 | 0.954 | 0.94 | 0.79 | 0.785 | 0.921 | 0.923 | 0.924 | ID | 0.927 | 0.901 | 0.933 | 0.927 | 0.927 | 0.789 | 0.947 | 0.929 | 0.929 |
| GZ70 | 0.921 | 0.928 | 0.932 | 0.924 | 0.923 | 0.941 | 0.934 | 0.933 | 0.791 | 0.778 | 0.929 | 0.928 | 0.989 | 0.927 | ID | 0.915 | 0.936 | 0.931 | 0.929 | 0.79 | 0.947 | 0.986 | 0.986 |
| MM93 | 0.914 | 0.925 | 0.931 | 0.915 | 0.927 | 0.912 | 0.914 | 0.918 | 0.785 | 0.769 | 0.927 | 0.928 | 0.91 | 0.901 | 0.915 | ID | 0.928 | 0.931 | 0.933 | 0.784 | 0.934 | 0.914 | 0.91 |
| MM53 | 0.928 | 0.938 | 0.947 | 0.927 | 0.944 | 0.962 | 0.942 | 0.964 | 0.791 | 0.789 | 0.942 | 0.941 | 0.936 | 0.933 | 0.936 | 0.928 | ID | 0.95 | 0.945 | 0.79 | 0.959 | 0.944 | 0.936 |
| MM19 | 0.938 | 0.947 | 0.951 | 0.931 | 0.947 | 0.934 | 0.938 | 0.938 | 0.798 | 0.782 | 0.949 | 0.95 | 0.931 | 0.927 | 0.931 | 0.931 | 0.95 | ID | 0.954 | 0.797 | 0.953 | 0.933 | 0.933 |
| GZ10 | 0.94 | 0.949 | 0.966 | 0.934 | 0.983 | 0.928 | 0.941 | 0.936 | 0.785 | 0.78 | 0.95 | 0.951 | 0.927 | 0.927 | 0.929 | 0.933 | 0.945 | 0.954 | ID | 0.784 | 0.953 | 0.934 | 0.929 |
| FJXM16 | 0.788 | 0.79 | 0.791 | 0.789 | 0.782 | 0.785 | 0.791 | 0.782 | 0.996 | 0.853 | 0.791 | 0.79 | 0.788 | 0.789 | 0.79 | 0.784 | 0.79 | 0.797 | 0.784 | ID | 0.79 | 0.799 | 0.794 |
| FJXM4 | 0.942 | 0.949 | 0.955 | 0.954 | 0.949 | 0.947 | 0.962 | 0.958 | 0.791 | 0.775 | 0.95 | 0.951 | 0.945 | 0.947 | 0.947 | 0.934 | 0.959 | 0.953 | 0.953 | 0.79 | ID | 0.947 | 0.945 |
| GZ56 | 0.924 | 0.931 | 0.94 | 0.929 | 0.928 | 0.941 | 0.937 | 0.936 | 0.801 | 0.788 | 0.932 | 0.931 | 0.984 | 0.929 | 0.986 | 0.914 | 0.944 | 0.933 | 0.934 | 0.799 | 0.947 | ID | 0.984 |
| GZ95 | 0.919 | 0.925 | 0.932 | 0.921 | 0.923 | 0.938 | 0.932 | 0.931 | 0.795 | 0.782 | 0.927 | 0.925 | 0.984 | 0.929 | 0.986 | 0.91 | 0.936 | 0.933 | 0.929 | 0.794 | 0.945 | 0.984 | ID |

Figure S1


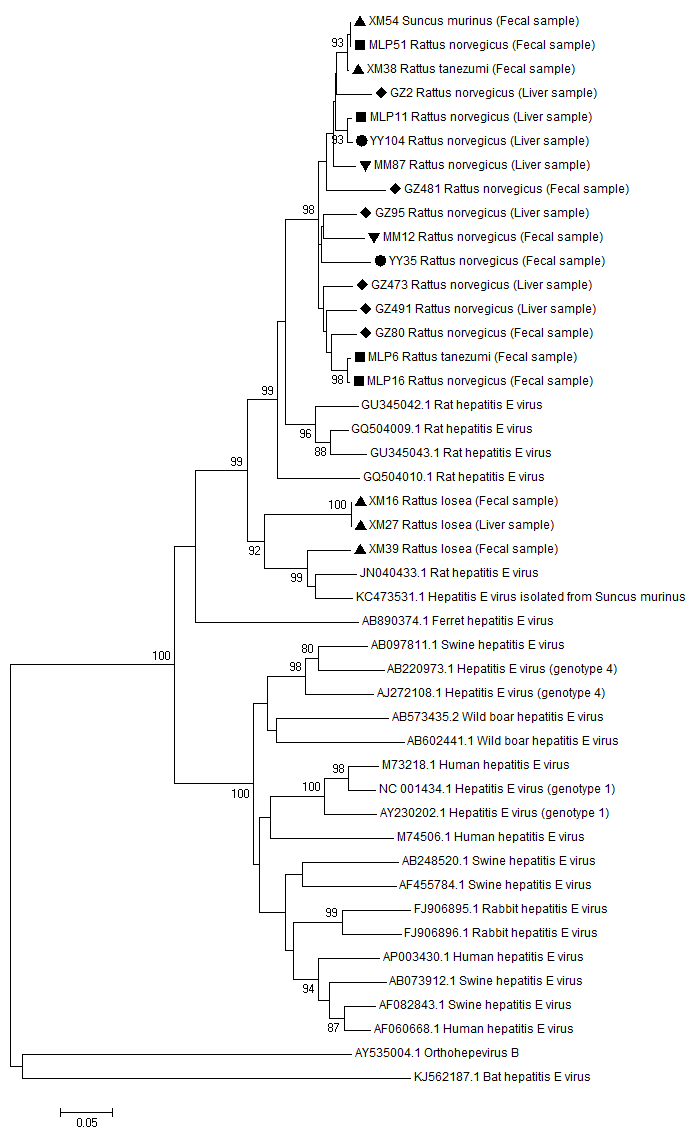


. XM, Xiamen City in Fujian Province; MLP, Malipo County in Yunnan Province; Guangzhou City in Guangdong Province; YY, Yiyang City in Hunan Province; MM, Maoming City in Guangdong Province.

Figure S2


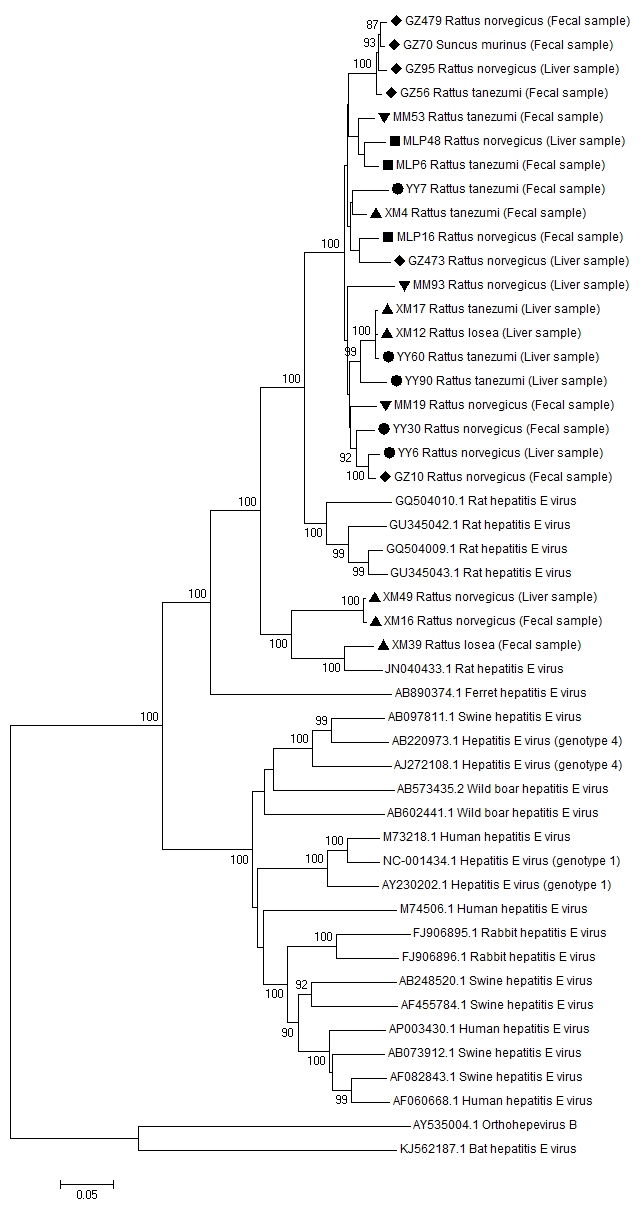


Guangzhou City in Guangdong Province; MM, Maoming City in Guangdong province;

MLP, Malipo County in Yunnan Province; YY, Yiyang City in Hunan Province; XM, Xiamen City in Fujian Province.

Figure S3


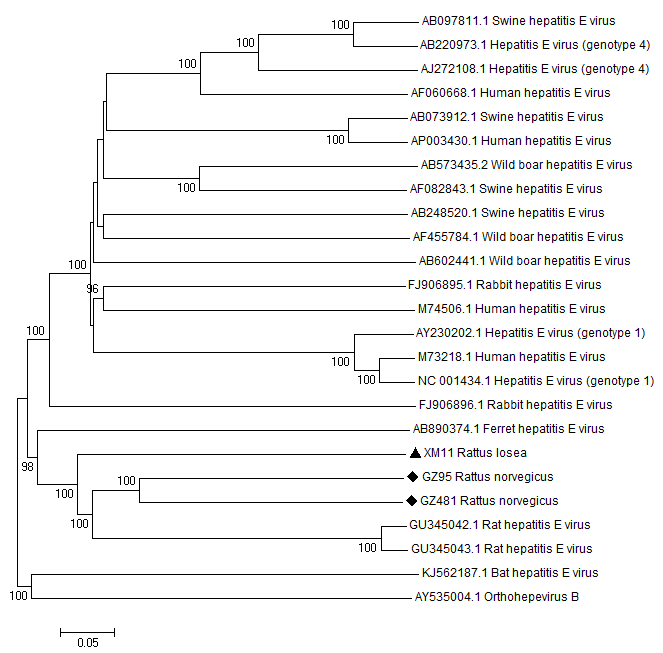


Guangzhou City in Guangdong Province; XM, Xiamen City in Fujian Province.

Figure S4


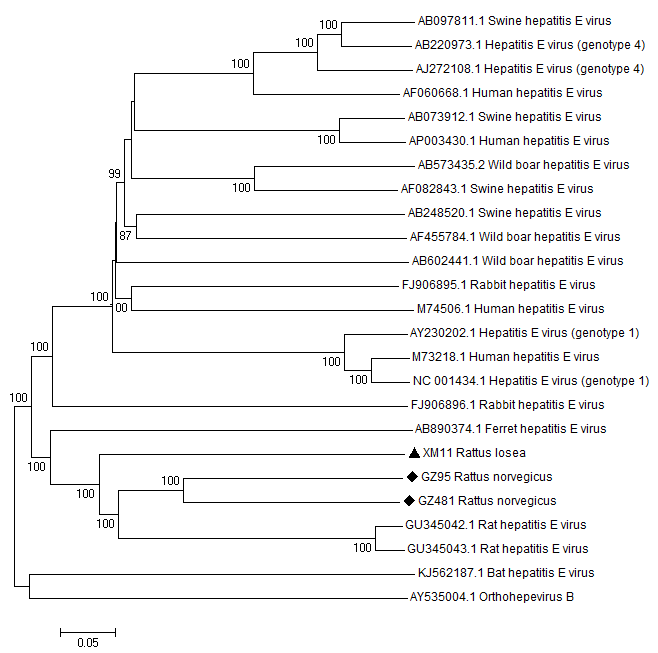


Guangzhou City in Guangdong Province; XM, Xiamen City in Fujian Province.

Figure S5


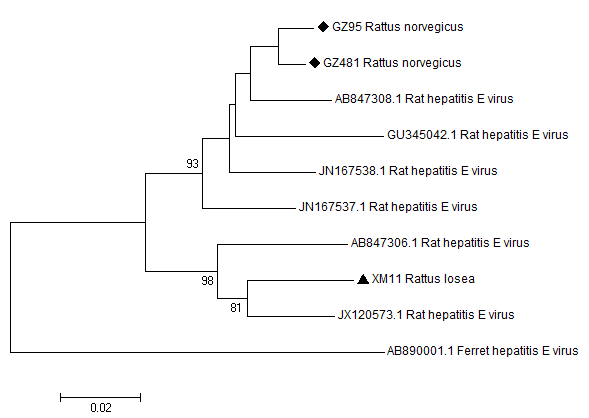


Guangzhou City in Guangdong Province; XM, Xiamen City in Fujian Province.

Figure S6


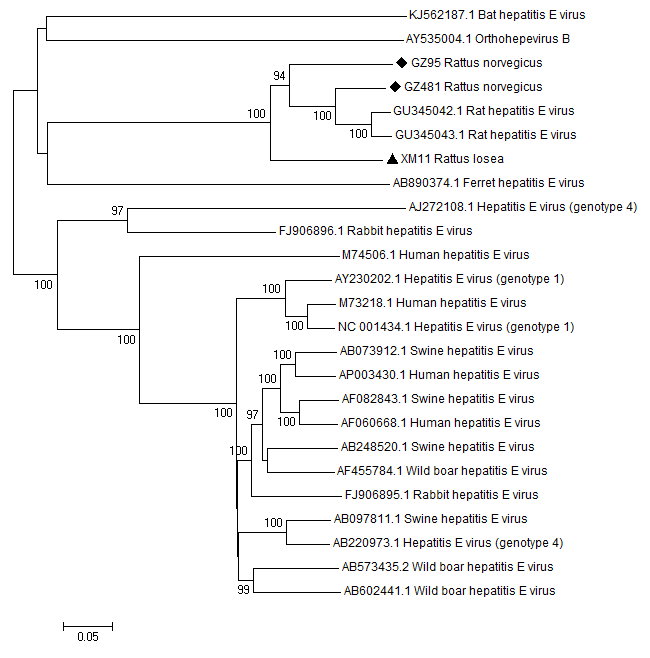


Guangzhou City in Guangdong Province; XM, Xiamen City in Fujian Province.

Figure S7


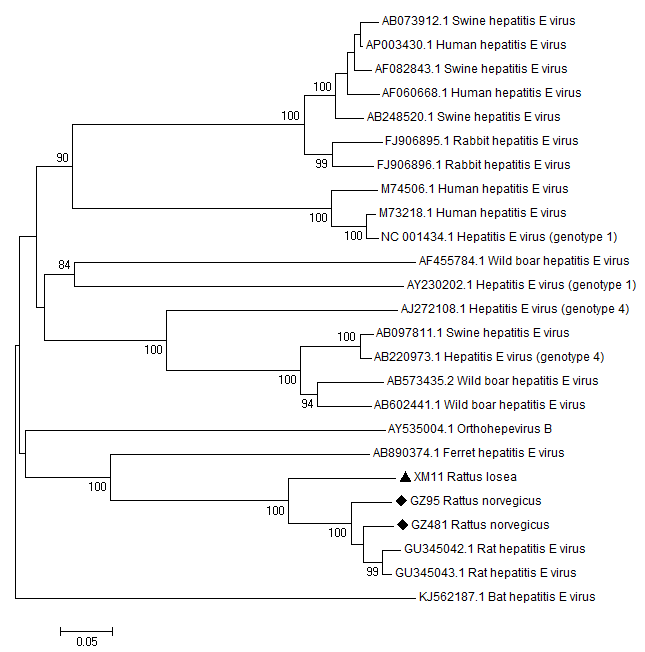


Guangzhou City in Guangdong Province; XM, Xiamen City in Fujian Province.
